# Compounded Disturbance Chronology Modulates the Resilience of Soil Microbial Communities and N-Cycle Related Functions

**DOI:** 10.3389/fmicb.2018.02721

**Published:** 2018-11-06

**Authors:** Kadiya Calderón, Laurent Philippot, Florian Bizouard, Marie-Christine Breuil, David Bru, Aymé Spor

**Affiliations:** ^1^Agroécologie, AgroSup Dijon, INRA, Université Bourgogne Franche-Comté, Dijon, France; ^2^Departamento de Investigaciones Científicas y Tecnológicas Universidad de Sonora, Hermosillo, Mexico

**Keywords:** compounded disturbances, nitrogen cycling, community composition, diversity, resilience

## Abstract

There is a growing interest of overcoming the uncertainty related to the cumulative impacts of multiple disturbances of different nature in all ecosystems. With global change leading to acute environmental disturbances, recent studies demonstrated a significant increase in the possible number of interactions between disturbances that can generate complex, non-additive effects on ecosystems functioning. However, how the chronology of disturbances can affect ecosystems functioning is unknown even though there is increasing evidence that community assembly history dictates ecosystems functioning. Here, we experimentally examined the importance of the disturbances chronology in modulating the resilience of soil microbial communities and N-cycle related functions. We studied the impact of 3-way combinations of global change related disturbances on total bacterial diversity and composition, on the abundance of N-cycle related guilds and on N-cycle related activities in soil microcosms. The model pulse disturbances, i.e., short-term ceasing disturbances studied were heat, freeze-thaw and anaerobic cycles. We determined that repeated disturbances of the same nature can either lead to the resilience or to shifts in N-cycle related functions concomitant with diversity loss. When considering disturbances of different nature, we demonstrated that the chronology of compounded disturbances impacting an ecosystem determines the aggregated impact on ecosystem properties and functions. Thus, after 3 weeks the impact of the ‘anoxia/heat/freeze-thaw’ sequence was almost two times stronger than that of the ‘heat/anoxia/freeze-thaw’ sequence. Finally, we showed that about 29% of the observed variance in ecosystem aggregated impact caused by series of disturbances could be attributed to changes in the microbial community composition measured by weighted UniFrac distances. This indicates that surveying changes in bacterial community composition can help predict the strength of the impact of compounded disturbances on N-related functions and properties.

## Introduction

All ecosystems are exposed to increasing natural and human disturbances and therefore understanding the effects of disturbances is fundamental. Most studies addressing ecosystem stability have included only one of numerous potential disturbances. For instance, the responses of ecosystems, plants and soil microbial communities to elevated CO_2_ or to changes in precipitation patterns have been the subject of major research efforts (Easterling et al., 2000). A rapidly growing number of studies have taken a more comprehensive approach to investigating interactive and cumulative effects between multiple co-occurring disturbances such as elevated CO_2_ and warming (Crain et al., 2008; Wu et al., 2011; Liang and Balser, 2012). However, while there is some generality in our understanding of the effects of single or simultaneous disturbances, the consequences of temporal series of disturbances for ecosystem functioning and stability are unclear. As the research front on the effects of human-driven environmental changes on ecosystems advances, it is now a timely challenge to anticipate the dynamics of the ecosystems response to multiple sequential disturbances (O’Gorman et al., 2012).

Disturbances are a strong driver of ecosystem dynamics over time. Whether the disturbance is of single or multiple nature, it is the ecosystem response to the series of disturbances that shapes its adaptive trajectory over time and hence the stability of its functioning. Because of their central role in Earth’s biogeochemical cycles (Falkowski et al., 2008), the effects of repeated disturbances of same nature, e.g., pollution or drought episodes on microbial communities have been investigated in several studies, often in relation to pollution induced community tolerance after exposure to heavy metals (Wakelin et al., 2014; Azarbad et al., 2016). These studies either revealed increased ecosystem adaptability (Bouskill et al., 2013) or ecosystem disruption (Shade et al., 2012), highlighting that history of disturbance regimes may play a role in responses of microbial communities toward new disturbances. However, ecosystems are also facing sequences of disturbances of multiple natures and above mentioned studies appear to be of limited value for predicting ecosystem response to a new or any type of disturbance. Series of disturbances of different nature have been considered when assessing whether a first disturbance could mediate the ecosystem response to a subsequent second disturbance (Crain et al., 2008; Darling and Côte, 2008; Philippot et al., 2008; Jackson et al., 2016; Jurburg et al., 2017). Although no clear overall pattern of response for the stability of soil microbial communities has emerged from such studies (Griffiths and Philippot, 2013), this approach could undeniably contribute to improve understanding of soil functioning. However, studies assessing the importance of the sequence in a succession of disturbances (i.e., disturbance chronology) are still lacking.

Here, we examine whether the responses of soil microbial community structure and function differ when subjected to the same series of disturbances but in different chronological order. Indeed, a disturbance B occurring after a disturbance A may very well displace the ecosystem to a different position on the adaptive trajectory, compared to its position if it had been exposed to these two disturbances in the reverse order. Therefore, we hypothesize that the disturbance chronology would affect ecosystem stability in terms of resistance (i.e., the ability to withstand a disturbance) and resilience (i.e., the capacity to recover after being disturbed). We studied the multiple functional responses of a soil system to sequences of three disturbances using heat, freeze-thaw and anaerobic cycles as model pulse disturbances, i.e., short-term ceasing disturbances. We focused on nitrogen cycling as an ecosystem function because nitrogen is the major nutrient limiting primary production in terrestrial ecosystems (LeBauer and Treseder, 2008). Among Earth-system processes, the nitrogen cycle is also one which was pushed by human activities outside critical thresholds representing the safe operating space (Rockström et al., 2009).

## Materials and Methods

### Soil Sampling and Experimental Design

Soil samples were collected from the Epoisses site in France (47° 30′ 22.1832′′ N, 4° 10′ 26.4648′′ E) during autumn 2014. During summer 2014, 12 days with temperature > 30°C were counted but the average summer temperature that year was relatively close to the average summer temperature in that area. Finally, winter and summer were rainier than average that year while spring was drier than average. Given the clayey nature of the soil, an increase in the precipitation amounts compared to normal might have caused anoxic conditions in the field. Soil properties were clay, 43.6%; sand, 14.3%; silt, 33.1%; organic carbon, 0.14%; organic nitrogen, 0.12% and pH 5.6. At each sampling site, soil was collected from four locations ca. 20 m apart from one another, by pooling 5 soil cores (20 cm depth) from 1 m × 1 m area at each location. All following steps were conducted by keeping the four replicate samples independent. All soils were sieved to 4 mm. 144 plasma flasks filled with 100 g of soil were then closed with sterile lids allowing gas exchanges between the atmosphere of the flask and atmospheric air. The microcosms were incubated at 20°C and regularly opened in a sterile laminar flow hood when adjusting the water holding capacity (WHC) between 60 and 80%.

### Disturbances Sequences

We considered three qualitatively different disturbances: freeze-thaw (-20°C; F), heat-drought (42°C; H) and anoxia cycles (A). These disturbances were chosen as model disturbances of interest because they have been used in multiple studies in microbial ecology (Sharma et al., 2006; Yergeau and Kowalchuk, 2008; Griffiths and Philippot, 2013). Soil microcosms (*n* = 120) were subjected to disturbance cycles consisting in two periods of 30 h of a given disturbance separated by a 40 h interval at 20°C. For the heat-drought disturbance, microcosms were placed into an incubator at 42°C; for the 20°C disturbance, microcosms were placed into a -20°C cold room and for the anoxia disturbance, oxygen was removed from the microcosms using a gas pump. Each disturbance cycle was then followed by 3 weeks incubation at 20°C with a maintained WHC ranging between 60 and 80% for all microcosms (Supplementary Figure S1). Note that soil humidity was monitored over the course of the experiment to allow us to control the WHC whatever the disturbance regime of the microcosms. Three cycles of disturbances were performed with either repeated disturbances of a same nature or compounded disturbances of different nature in every possible order (*n* = 4). Control microcosms were incubated in the same conditions without being exposed to disturbance cycles, which resulted in a total of 140 microcosms. We destructively sampled microcosms at day 9 (T_0_: before any disturbance), day 36 (T_1_: 3 weeks after the 1st cycle of disturbance), day 64 (T_2_: 3 weeks after the 2nd cycle of disturbance), day 92 (T_3_: 3 weeks after the 3rd cycle of disturbance) and day 148 (T_4_: 10 weeks after the 3rd cycle of disturbance). Note that measurements over time are independent because at each time point, different microcosms were sampled.

### Nitrogen Pools

Mineral nitrogen pools (NO_3_^-^ and NH_4_^+^) present in soil were extracted using 50 ml of KCl 1M that was added to ca. 10 g fresh soil, shaken vigorously (80 rpm for 1 h at room temperature), filtered and kept frozen until quantification according to ISO standard 14256-2 (Calderón et al., 2017). Quantification was performed using at least two blanks in each series by colorimetry in a BPC global 240 photometer.

### Potential Nitrification Activity (PNA)

Potential nitrification activity (PNA) was performed according to ISO 15685. Briefly, 1.4 mM sulfate ammonium was added to 10 g of fresh weight soil supplemented with 500 mM of sodium chlorate to block the oxidation of nitrite. Ammonium oxidation rates were determined in each sample by measuring the accumulated nitrite every 2 h during 6 h via a colorimetric assay (Kandeler, 1995).

### Potential Denitrification Activity (PDA) and Potential N_2_O Emissions

Potential denitrification activity (PDA) (N_2_O + N_2_) and potential nitrous oxide emissions (N_2_O) were measured using the acetylene inhibition technique (Yoshinari and Knowles, 1976). For each sample, 10 g of fresh weight soil was wetted with 20 ml of distilled water and was amended with a final concentration of 3 mM KNO3, 1.5 mM succinate, 1 mM glucose, and 3 mM acetate. To determine the potential denitrification activity, acetylene was added to reach 0.1 atm partial pressure followed by 30 min incubation at 25°C and agitation (175 rpm). Gas samples were taken every 30 min for 150 min (Pell et al., 1996). The N_2_O concentrations were determined using a gas chromatograph (Trace GC Ultra, Thermo Scientific) equipped with an EC-detector.

### Quantification of Microbial Communities

DNA was extracted from 250 mg dry-weight soil samples according to ISO standard 11063 “Soil quality-Method to directly extract DNA from soil samples” (Petric et al., 2011). Total bacterial communities were quantified using 16S rRNA primer-based qPCR assays, respectively, (Muyzer et al., 1993; Ochsenreiter et al., 2003). Quantification of the bacterial and archaeal ammonia-oxidizers (AOB and AOA, respectively) was performed according to Leininger et al. (2006) and Tourna et al. (2008) whereas quantification of denitrifiers was performed according to Henry et al. (2004, 2006) and Jones et al. (2013). For this purpose, the genes encoding catalytic enzymes of ammonia-oxidation (bacterial and archaeal *amoA*), of nitrite reduction (*nirK* and *nirS*) and of nitrous oxide reduction (*nosZI* and *nosZII*) were used as molecular markers. Although not covering the extent genetic diversity of each group, the *nirS* and *nirK* primer sets used still allow for a comparative analysis of the relative abundance of each across the different soils samples by sampling a standard subset of each group for which denitrification functionality is verified (Penton et al., 2013). Reactions were carried out in a ViiA7 (Life Technologies, United States). Quantification was based on the increasing fluorescence intensity of the SYBR Green dye during amplification. The real-time PCR assays were carried out in a 15 μl reaction volume containing SYBR green PCR Master Mix (Absolute Blue QPCR SYBR Green Low Rox Mix, Thermo, France), 1 μM of each primer, 250 ng of T4 gene 32 (QBiogene, France) and 0.5 ng of DNA as previously described (Bru et al., 2013). Three independent replicates were used for each real time PCR assay. Standard curves were obtained using serial dilutions of linearized plasmids containing appropriated cloned targeted genes from bacterial strains or environmental clones. PCR efficiency for the different assays ranged from 70 to 99%. No template controls gave null or negligible values. The presence of PCR inhibitors in DNA extracted from soil was estimated by mixing a known amount of standard DNA with soil DNA extract prior to qPCR. No inhibition was detected in any case. qPCR data are presented in number of copies of a given gene per ng DNA.

### Assessment of Microbial Community Composition and Diversity

A 2-step PCR approach was used for amplification of the V3–V4 hypervariable region of the 16S rRNA gene according to Berry et al. (2011). The first step was run on three subsamples that were subsequently pooled. It consisted of 20 μM of the forward primer 515F 5′-GTGCCAGCMGCCGCGGTAA-3′, 20 μM of the reverse primer 806R 5′-GGACTACHVGGGTWTCTAAT-3′ (Eurogentec Seraing, Belgium), together with 10X buffer with MgSO_4_ (Promega), 1U Pfu DNA polymerase, 20 μM dNTPs (MP Biomedicals, Europe), 250 ng T4 gp32 bacteriophage (MP Biomedicals, Europe) and 50 ng DNA template in a final volume of 25 μL. Reaction conditions were as follows: 2 min at 95°C followed by 20 cycles of 30 s at 95°C, 30 s at 53°C and 60 s at 72°C on an MJ Research PTC-200 Thermal Cycler (Bio-Rad, CA, United States). In the second step, 1 uL of the pooled PCR products of the first step was amplified in triplicate in a 10-cycle PCR using the forward primers preceded by 10 basepair-long barcodes, the sequencing key and the forward sequencing adapter; the reverse primers being preceded by the sequencing key and the reverse sequencing adapter only. The final PCR products were pooled and extracted from 2% agarose gel with the QIAEX II kit (Qiagen; France) and finally quantified using the Quant-iT PicoGreen dsDNA Assay Kit (Invitrogen, Cergy-Pontoise, France). Pyrosequencing was performed by Genoscreen sequencing service (Lille, France) on a Roche 454 FLX Genome Sequencer using Titanium chemistry (Roche Diagnostics).

### Bioinformatic Analysis of the 16S rRNA Amplicons

The sequences obtained were analyzed using QIIME pipeline software (Caporaso et al., 2010b). Sequences of poor quality (score < 25 on a 50 base pair sliding window) or shorter than 230 base pairs were removed. Reference-based chimera detection was performed using greengene’s representative set of 16S rRNA sequences and 1,297,290 quality-filtered reads were clustered in Operational Taxonomy Units (OTUs) at 97% similarity using USEARCH (Edgar, 2010). Representative sequences for each OTU (6394 OTUs retrieved) were then aligned using PyNAST (Caporaso et al., 2010a) and their taxonomy assigned using the greengenes database^1^. No choloroplast or mitochondrial OTUs were retrieved in our dataset. A phylogenetic tree was then constructed using FastTree (Price et al., 2010). Raw sequences were deposited at the NCBI under the accession number SRP117152. The process of raw sequences submission was greatly simplified by using the *make.sra* command of Mothur software (Schloss et al., 2009).

Diversity metrics, i.e., Faith’s Phylogenetic Diversity (Faith, 1992), richness (observed species) and evenness (Simpson’s reciprocal index), describing the structure of microbial communities were calculated based on OTU tables that were rarefied to 2200 sequences per sample, corresponding to the minimum number of sequences in a given sample, from which singletons were removed. Unweighted and weighted UniFrac distance matrices (Lozupone et al., 2010) were also computed to detect global variations in the composition of microbial communities. Principal Coordinates Analyses (PCoA) were then calculated and plotted. Discriminant OTUs between control and disturbed microcosms were detected using the pamr package (Wood et al., 2007).

### Statistical Analyses

All statistical analyses were performed in R Studio (version 3.0.2) using the following R packages: vegan (Oksanen et al., 2016), RcolowBrewer (Neuwirth, 2014), gplots (Warnes et al., 2016), and car (Fox et al., 2016). Differences in gene copy abundance (16S rRNA, bacterial and archaeal *amoA*, *nirK* and *nirS*, *nosZ*I, and *nosZ*II), total nitrogen, ammonium and nitrate concentrations, and α-diversity indexes were tested using ANOVAs at each timepoint with the following model: *Y*_ij_ = *μ* + treatment_i_ + residual_ij_, followed by Tukey HSD tests. Normality and homogeneity of the residuals distribution was inspected and log-transformations were performed when necessary. Variance partitioning techniques were used to explain variations of different nitrogen pools (i.e., ammonium or nitrate) by variations of N-cycle microbial activities (nitrification, potential N_2_O emissions, potential denitrification and N_2_O emission ratio), variations of the abundance of different N-cycle microbial guilds (16S rRNA, AOA and AOB, *nirK* and *nirS*, *nosZI*, and *nosZII*), and variations of the total microbial diversity (observed species, Faith’s PD and Simpson’s reciprocal indices).

### Ecosystem Aggregated Impact

We calculated effect sizes, and their respective 95% confidence intervals, using Hedges’ g, an estimate of the standardized mean difference not biased by small sample sizes (Gurevitch et al., 2001) that is classically used in ecology to quantify effects of disturbances on ecosystem properties, for each variable comparing control and treated samples. We calculated an “Ecosystem Aggregated Impact” as the sum of the absolute value of Hedges’ g for the 26 studied variables (List A, Supp Mat). Note that we chose to take the sum of absolute value because we did not want to have subjective *a priori* regarding what could be a better or worse performance for a given variable. The objective of this study was not to determine whether or not ecosystem performances of disturbed microcosms are better or worse than the control ones, but to quantify how much it changed. The corresponding variance was calculated as the sum of the variance of each variable Hedges’ g, which is valid for approximately normally distributed variables. Non-overlapping 95% confidence intervals were then considered as significantly different.

## Results and Discussion

### Resilience or Global Drift of Microbial Communities and N-Related Functions After Repeated Disturbances of the Same Nature

**FIGURE 1:**
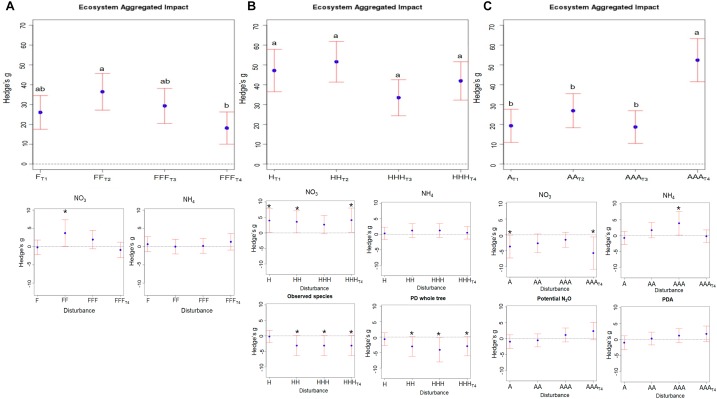
Aggregated impact of repeated disturbances on ecosystem properties and functions. The “Ecosystem Aggregated Impact” was calculated as the sum of the absolute value of Hedges’ g for the 26 studied variables. The corresponding variance is the sum of the variance of each variable Hedges’ g. 95% confidence intervals are represented for each treatment (**A**) corresponds to the Freeze-Thaw disturbances: F; Panel **B** to the Heat disturbance: H; and panel **C** to the Anoxia disturbance: A. In each panel, the corresponding treatment effects on ammonium and nitrate pools as well as on chosen ecosystem properties and functions is given. Note that the control treatment cannot be plotted on this figure because each ecosystem functions and properties (EAI) is calculated as an effect size of a given treatment relative to the control. Different letters above the bars indicate significant differences.

We calculated an index describing the aggregated measures of the impact of disturbances on ecosystem functions and properties (EAI). We observed three different patterns of EAI depending on the nature of the disturbances. When facing a series of freeze-thaw disturbances, the measured functions and properties showed an overall tendency to resilience, defined here as a relative proximity to control microcosms, with significantly decreasing EAI values from T_2_ [EAI = 36.5; CI_95%_ = (27.2 – 45.7)] to T_4_ [EAI = 18.2; CI_95%_ = (10.1 – 26.3)] (Figure 1A). We detected a significant and transient NO_3_^-^ accumulation in disturbed microcosms compared to control ones (Figure 1A). These shifts in NO_3_^-^ pools were significantly positively related to shifts in the abundance of ammonia oxidizing archaea and to the relative proportion of ammonia oxidizing bacteria, but negatively related to shifts in PDA (Supplementary Table S1). This indicates that denitrification was temporarily slowed down by repeated freeze-thaw disturbances but recovered after 10 weeks, while the disturbances did not impact nitrification over the course of the experiment as shown by the absence of difference at all time points between control and disturbed microcosms (Figure 1A). This supports previous findings reporting that freeze-thaw cycles had no inhibitory effect on the nitrification potential in several different soils (Yanai et al., 2004).

Heat disturbances caused relatively strong modifications of soil functions and properties [33.5; CI_95%_ = (24.3 – 42.7)] < EAI < 51.6; CI_95%_ = (41.3 – 61.9)] (Figure 1B) that remained over time (no significant differences between EAI values at T_1_, T_2_, T_3_, and T_4_). We found a significant accumulation of NO_3_^-^ in heat-disturbed microcosms compared to control ones during the course of the experiment. PDA decreased significantly in disturbed compared to control microcosms and was more heavily impacted than potential N_2_O emissions, especially at T_3_ and T_4_. This decrease in potential N_2_O emissions was negatively correlated to NO_3_^-^ accumulation. These results parallel those from previous studies highlighting alteration of microbial processes such as N-cycling in response to a heat disturbance (Mooshammer et al., 2017). We also found that repeated heat disturbances was the only series of the same disturbance treatment having an impact on bacterial diversity with a significant decrease of phylogenetic diversity and richness in disturbed microcosms at T_2_, T_3_, and T_4_, associated with NO_3_^-^ accumulation (Supplementary Table S1 and Figure 1B). Former studies showing weak relationships between microbial diversity and ecosystem functions, suggested that functional redundancy, i.e., the ability of different species to perform similar roles under the same environmental conditions, was important in microbial communities (Wertz et al., 2007; Schimel and Schaeffer, 2012; Philippot et al., 2013). In contrast, we found that the modifications in N-cycling were related to a significant decrease of bacterial richness in disturbed microcosms, which challenges the extent of functional redundancy. However, the degree of redundancy within different microbial functional groups is still hotly debated since divergent controversial results are reported (Bell et al., 2005; Allison and Martiny, 2008; Strickland et al., 2009; Calderón et al., 2017).

We observed an apparent short-term resistance of ecosystem properties and functions to repeated anoxia disturbances [relatively constant EAI over T_1_: EAI = 19.3; CI_95%_ = (10.9 – 27.6), T_2_: EAI = 26.9; CI_95%_ = (18.3 – 35.5) and T_3_: EAI = 18.7; CI_95%_ = (10.4 – 26.9)] (Figure 1C). However, this period of relative stability was followed by a strong and significant alterations of soil properties and functions after 10 weeks [EAI = 52.4; CI_95%_ = (41.5 – 63.3) at T_4_] (Figure 1C). Thus, larger modifications in N-cycling were observed at T_4_ with increased PDA and potential N_2_O emissions (Figure 1C). This stimulation of denitrification – a facultative respiratory process during which nitrate is reduced into gaseous nitrogen when oxygen is limited – in microcosms exposed to anoxia, and the concomitant depletion of both NO_3_^-^ and total N (Supplementary Table S1), was largely expected. Interestingly, we also observed a transient NH_4_^+^ accumulation after repeated anoxia cycles, especially at T_3_ that was not related to a change in potential nitrification, nor to a change in the abundance of AOA or AOB although the abundance of AOA decreased at T_4_.

Overall, these results suggest that depending on the nature of the disturbance, repeated environmental disturbances can lead either to a resilience of soil properties and functions once the disturbance ceases or to a shift in soil properties and functions indicating that a number of repeated pulse disturbances can gradually impair the ecosystem capacity to sustain its domain of stability (Villnas et al., 2013). However, not only disturbance frequency but also disturbance intensity can alter ecosystem stability (Berga et al., 2012). Because it is not feasible to assess differences in intensity between various types of disturbances, the effects of the nature of disturbance and of its intensity are therefore intrinsically linked in our study.

### The Chronology of Compounded Disturbances Impacting Soil Ecosystems Determines Microbial Community Composition as Well as Ecosystem Properties and Functions

**FIGURE 2:**
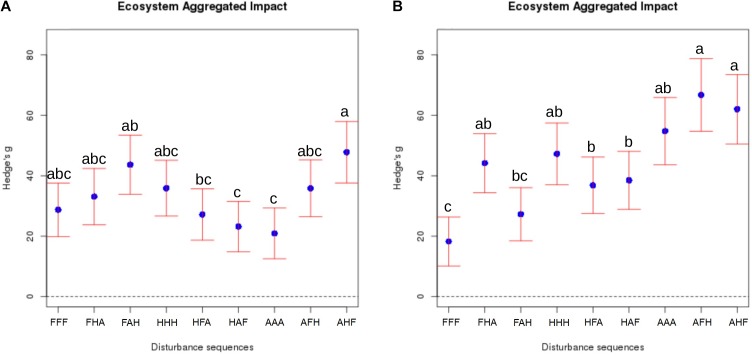
Aggregated impact of compounded disturbances with alternative chronologies on ecosystem properties and functions. The “Ecosystem Aggregated Impact” was calculated as the sum of the absolute value of Hedges’ g for all studied variables. The corresponding variance is the sum of the variance of each variable Hedges’ g. 95% confidence intervals are represented for each treatment. Panel **A** corresponds to T_3_ and panel **B** to T_4_, Disturbance sequences are encoded with F: Freeze-thaw, H: Heat and A: Anoxia. Note that the control treatment cannot be plotted on this figure because each EAI is calculated as an effect size of a given treatment relative to the control. Different letters above the bars indicate significant differences.

After applying the selected disturbances in every possible order, the aggregated impact of compounded disturbances on ecosystem properties and functions was calculated as described above. Results of these analyses revealed a significant effect of the chronology of disturbances on the EAI, which supports our hypothesis (Figure 2). This was particularly obvious for the ‘anoxia/heat/freeze-thaw’ sequence, whose impact after 3 weeks (T_3_) was almost two times stronger [EAI = 47.8 (37.6 – 57.9)] than that of the ‘heat/anoxia/freeze-thaw’ sequence [EAI = 23.2 (14.9 – 35.7)]. A striking result of our study is that differences between sequences of disturbances were even more pronounced at T_4_ than T_3_, with the ‘anoxia/freeze-thaw/heat’ sequence having the strongest impact [EAI = 66.8 (54.7 – 78.8)] and the ‘freeze-thaw/anoxia/heat’ one being the less disturbing [EAI = 27.3 (18.4 – 36.1)]. While the idea that microbial communities display a high resistance and resilience is pervasive in ecology (Allison and Martiny, 2008), our results suggest instead a poor resilience since legacy effects of compounded disturbances are increasing with time. Because 10 weeks elapsed after the last disturbance, it is not possible to decipher whether these differences observed at T_4_ are still increasing of if they had reached a plateau somewhere in between the two sampling events.

**FIGURE 3:**
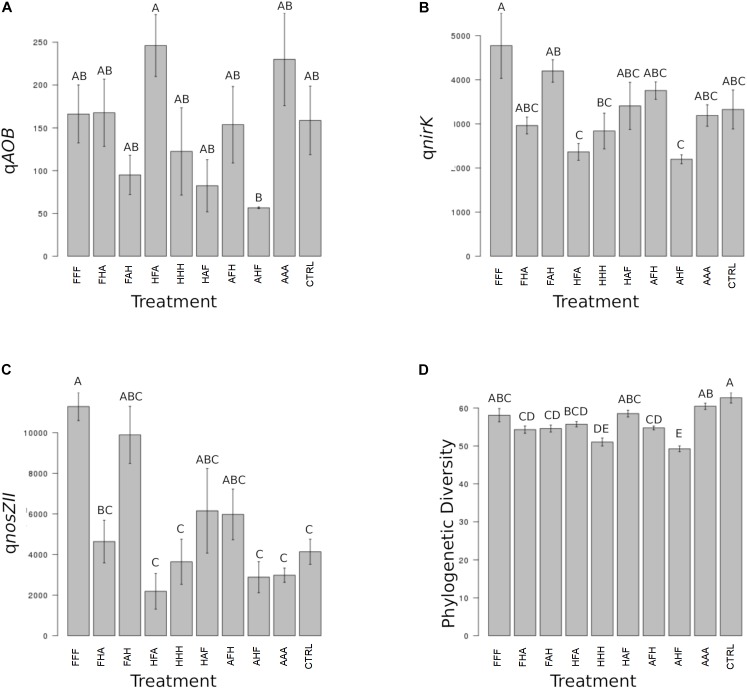
Ecosystem properties significantly impacted by the chronology of compounded disturbance at T_3_. Different letters above the bars indicate significant differences according to Tukey’s test (*p* < 0.05). **(A)** qAOB corresponds to the abundance of ammonia-oxidizing bacteria and is expressed in gene copies x g^-1^ DNA. **(B)** qnirK corresponds to the abundance of bacteria harboring the *nirK* nitrite reductase gene and is expressed in gene copies x g^-1^ DNA. **(C)** qnosZII corresponds to the abundance of bacteria harboring the *nosZ* clade II N_2_O reductase gene and is expressed in gene copies x g^-1^ DNA. **(D)** Phylogenetic Diversity corresponds to estimates of the Faith’s phylogenetic diversity index.

**FIGURE 4:**
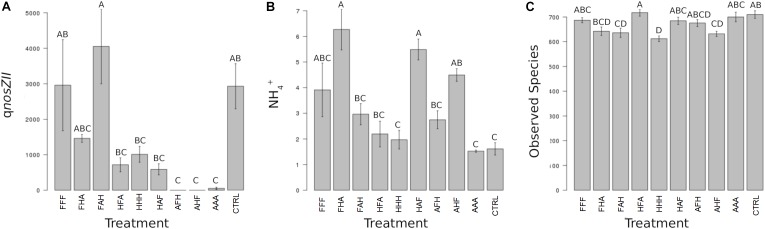
Ecosystem properties significantly impacted by the chronology of compounded disturbance at T_4_. Different letters above the bars indicate significant differences according to Tukey’s test (*p* < 0.05). **(A)** qnosZII corresponds to the abundance of bacteria harboring the *nosZ* clade II N_2_O reductase gene and is expressed in gene copies x g^-1^ DNA. **(B)** NH_4_^+^ corresponds to soil NH_4_^+^ pool sizes and is expressed in mg N x kg^-1^ DNA. **(C)** Observed Species corresponds to the species richness observed in treated and control microcosms and is expressed in number of species.

When considering individual variables separately, we identified the abundance of AOB, *nirK*-denitrifiers (at T_3_) and of the *nosZ*II clade (at T_3_ and T_4_) as significantly impacted by the disturbance chronology (Figures 3C, 4A). Regarding AOBs, estimates were up to five times lower for the ‘anoxia/heat/freeze-thaw’ sequence (AHF) comparing to the ‘heat/freeze-thaw/anoxia’ one (HFA) at T_3_ (Figure 3A). Accordingly, Wessen and Hallin (2011) proposed that the abundance of ammonia-oxidizers could be a good bioindicator for soil monitoring while the composition of this guild was suggested among the possible ecologically relevant biological indicators of soil quality (Ritz et al., 2009). For both *nirK*- and *nosZII*-communities at T_3_, we found their maximum abundances in the ‘freeze-thaw/anoxia/heat’ sequence (FAH), while their abundances were significantly lower in the AHF (Figures 3B,C). At T_4_, the *nosZ*II community was undetectable in two of the chronology treatments (the ones starting with the ‘anoxia’ disturbance) while its abundance in the FAH remained in the same range than that of the control treatment (Figure 4A). Not only the abundance of microbial guilds involved in N-cycling but also N-pools were affected by disturbance chronology. In particular, the NH_4_^+^ concentration was about three times higher in the ‘freeze-thaw/heat/anoxia’ sequence (FHA) than in the HFA (Figure 4B). However, disturbance chronology had no effect on the measured processes and the disturbance sequences impacting significantly the abundance of N-cycling communities and the NH_4_^+^ pools were not the same. This adds fuel to the debate about the links between functional community abundances and corresponding process rates and/or pools of products (Rocca et al., 2015; Graham et al., 2016).

**FIGURE 5:**
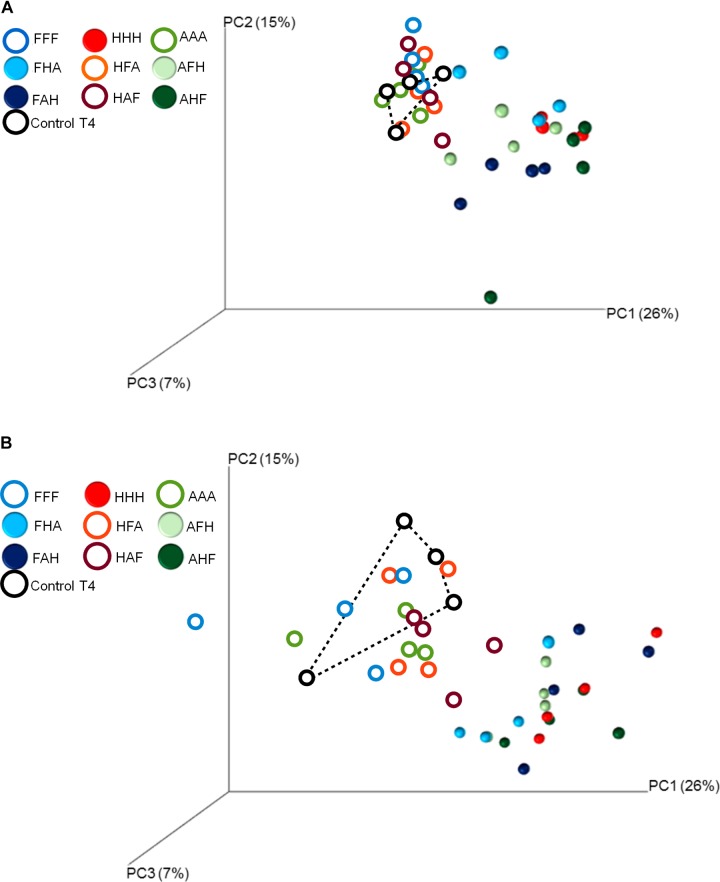
Principal Coordinates Analyses of the weighted UniFrac distance matrix representing differences in community structure between control and treated microcosms at T_3_ **(A)** and T_4_ **(B)**. Different colors correspond to the different treatments and to control microcosms. Closed symbols are used to show treatments that are significantly different from the control microcosms (pairwise-PERMANOVAs).

We also demonstrate that disturbance chronology caused significant shifts in bacterial phylogenetic diversity and richness at T_3_ but also at T_4_ (Figures 3D, 4C)_._ Such differences in bacterial community diversity at T_4_, 10 weeks after the last disturbance had occurred, highlight the importance of legacy effects of the disturbance chronology, which can overwhelm the short term effect of the last disturbance. This is exemplified by the FAH and the ‘anoxia/freeze-thaw/heat’ (AFH) sequences leading to significantly different EAI at T_4_ but not at T_3_ (Figure 2). The AHF sequence was detected as the one with the strongest impact on bacterial phylogenetic diversity and richness with losses up to ∼20% of the PD at T_3_ and ∼15% of the richness a T_4_ compared to control treatments (Figures 3D, 4C). In contrast, the ‘heat/anoxia/freeze-thaw’ sequence (HAF) for example did not display any significant diversity loss at both times. As expected, these changes in diversity levels due to the chronology of disturbances were most often concomitant with significant differences in bacterial community structure (measured as differences in weighted UniFrac distances, pairwise-PERMANOVAs) at T_3_ (Figure 5A) and T_4_ (Figure 5B). Altogether, these results highlight that the chronology of compounded disturbances impacts significantly the resilience of ecosystem properties and functions through modifications of the bacterial community structure, abundance and diversity. Consequently, historical information about the succession of disturbances is another element that would improve our understanding of patterns in microbial communities, which is particularly important in the context of global change leading to increasing extreme climatic events.

### Predicting the Strength of the Ecosystem Gggregated Impact of Series of Disturbances on Soil Properties and Functions

A significant part of the observed variance in EAI values caused by series of disturbances was explained by changes in total bacterial community structure and in N_2_O reducer abundances (Table 1). We found that ∼29% of the EAI variance could be attributed to changes in weighted UniFrac distances. This means that a significant part of the changes observed in ecosystem properties and functions can be linked to changes in bacterial community composition. This indicates that the prediction of the stability of aggregated ecosystem properties and functions after series of disturbances is possible, to some extent, based on measured changes of microbial community composition. The concept of functional redundancy is often used as a justification for considering community composition as less relevant for ecosystem processes, even when facing environmental disturbances (reviewed in Griffiths and Philippot, 2013 and Graham et al., 2016). For example, Wertz et al. (2006) found that decline in biodiversity did not impair either the resistance or resilience of two N-cycling guilds following a heat disturbance. On the contrary, our results indicate that the impact of compounded series of disturbances on soil properties and function is reflected by the degree of phylogenetic relatedness between microbial communities. This yields additional lines of evidence supporting the importance of microbial community composition and diversity for maintaining ecosystem functioning under fluctuating conditions (Yachi and Loreau, 1999; Fetzer et al., 2015). Moreover, our findings suggest that shifts in microbial community composition in disturbed environments can be a quantitative indicator of the degradation of the ecosystem functioning. We were also able to detect OTUs that were significantly enriched/depleted in control versus disturbed microcosms. While the sensitive-to-disturbances OTUs belong to diverse phyla (Supplementary Figure S2A), 4 out of 6 of the resistant-to-disturbances OTUs have been classified as members of the Actinobacteria phylum (Supplementary Figure S2B with two members of the Actinomycetales order and two members of the Thermoleophilia class). Besides the composition of the total bacterial community, we also detected shifts in N_2_O-reducer abundances (using the abundance of *nosZ*I and *nosZ*II genes as proxies) as indicative of changes in EAI values with, respectively, ∼35 and ∼13 % of explained variance for *nosZ*I and *nosZ*II-clade N_2_O reducers. This higher susceptibility of N_2_O-reducers to environmental changes makes the abundance of this guild an effective candidate marker of disturbances when considering N-cycle related ecosystem functions.

**Table 1:** Predicting the aggregated impact of compounded disturbances on ecosystem properties and functions.

Explaining variable	Mean square	*F*-value
*Weighted UniFrac distance*	1315.36 (29%)	54.83^∗∗∗^
*Potential Nitrification Activity*	92.63	3.86
*q*nosZI*/q*16S	1546.38 (35%)	64.45^∗∗∗^
*q*nosZII	623.36 (13%)	25.98^∗∗∗^
*q*nirK	92.47	3.85
*q*nirK*/q*16S	193.07	8.05^∗^
*Faith’s PD*	33.54	1.39
*Simpson’s reciprocal*	100.44	4.19
*Residuals*	23.99	

*Multiple linear regressions were used to assess the % of ecosystem functions and properties (EAI) variance explained by each explaining variables. Note that regarding the weighted UniFrac distance, we calculated the distance between each treatment centroid and its corresponding control centroid. Therefore we ended up with a vector of control-to-treatment distances that we used as an explaining variable in the multiple regression. % of EAI variance explained by each explaining variables is given between parentheses. Significance levels: ^∗∗∗^ < 0.001, ^∗∗^ < 0.01, ^∗^ < 0.05.*

Altogether, using model, controlled pulse disturbance sequences that are not necessarily environmentally relevant, our results demonstrate the non-commutative property of sequential environmental disturbances of a different nature. The chronological order in which disturbances are occurring can make a soil ecosystem increasingly vulnerable to subsequent disturbances due to legacy effects affecting durably soil microbial community composition. History of disturbances can therefore help us to elucidate the mechanisms underlying observed patterns in microbial communities. Ecosystems worldwide are experiencing higher pressures due to the combined and intricate effects of anthropogenic activities and climate change. Building a predictive framework of the impact of compounded disturbances on soil functioning strongly depends on the identification of ecological markers of disturbances for assessing ecosystems health in a context of sustainable land use. In this perspective, we show that the aggregated impact of series of disturbances on soil properties and functions were reflected by shifts in community composition, which suggest that assessing the stability of microbial communities can be an effective proxy for monitoring the ecosystem functional resilience to compounded disturbances. This also further emphasizes the benefit of incorporating microbes into ecosystem process models.

## Author Contributions

AS and LP conceived and designed the experiment. KC, DB, FB, and M-CB performed the experiments. AS, KC, and LP analyzed the data. AS and LP wrote the manuscript with the help of KC. All authors approved the manuscript.

## Conflict of Interest Statement

The authors declare that the research was conducted in the absence of any commercial or financial relationships that could be construed as a potential conflict of interest.

## Footnotes

1 http://greengenes.lbl.gov/Download/Sequence_Data/Greengenes_format/
