# Supplementary material for: Compounded Disturbance Chronology Modulates the Resilience of Soil Microbial Communities and N-Cycle Related Functions
Source: Front Microbiol. 2018 Nov 6;9:2721. doi: 10.3389/fmicb.2018.02721 (PMC6232425; doi:10.3389/fmicb.2018.02721)

Supplementary Materials :

Compounded disturbance chronology modulates the resilience of soil microbial communities and N-cycle related functions

Authors: Kadiya Calderón[1,2*], Laurent Philippot[1*], Florian Bizouard[1], Marie-Christine Breuil[1], David Bru[1] & Aymé Spor[1#]

Authors affiliation:

[1]*INRA, UMR 1347 Agroecology, Dijon, France*

[2]*Universidad de Sonora. Departamento de Investigaciones Científicas y Tecnológicas de la Universidad de Sonora. Blvd. Luis Donaldo Colosio S/N. Hermosillo, Sonora, Mexico.*

[*] These authors contributed equally to this manuscript

#Corresponding author: A. Spor, ayme.spor@inra.fr

Supplementary Figure 1. Experimental Setup.

Days	1	8	15	22	29	36	43	50	57	64	71	78	85	92	99	106	113	120	127	134	141	148
Sampling		4 Control t0				4 Control t1				4 Control t2				4 Control t3								4 Control t4
						4 Stress F t1				4 Stress F-F t2				4 Stress F-F-F t3								4 Stress F-F-F t4
						4 Stress H t1				4 Stress H-H t2				4 Stress H-H-H t3								4 Stress H-H-H t4
						4 Stress A t1				4 Stress A-A t2				4 Stress A-A-A t3								4 Stress A-A-A t4
										4 Stress F-H t2				4 Stress F-H-A t3								4 Stress F-H-A t4
										4 Stress F-A t2				4 Stress F-A-H t3								4 Stress F-A-H t4
										4 Stress H-F t2				4 Stress H-F-A t3								4 Stress H-F-A t4
										4 Stress H-A t2				4 Stress H-A-F t3								4 Stress H-A-F t4
										4 Stress A-F t2				4 Stress A-F-H t3								4 Stress A-F-H t4
										4 Stress A-H t2				4 Stress A-H-F t3								4 Stress A-H-F t4
Stress Application		**1st Stress Application**				**2nd Stress Application**				**3rd Stress Application**				**Short-Term Resilience**								**Long-Term Resilience**
		40 microcosms with F stress				36 microcosms with F stress				24 microcosms with F stress												
		40 microcosms with H stress				36 microcosms with H stress				24 microcosms with H stress												
		40 Microcosms with A stress				36 microcosms with A stress				24 microcosms with A stress												
**Microcosms left**		**136**				**120**				**80**				**40**								**0**

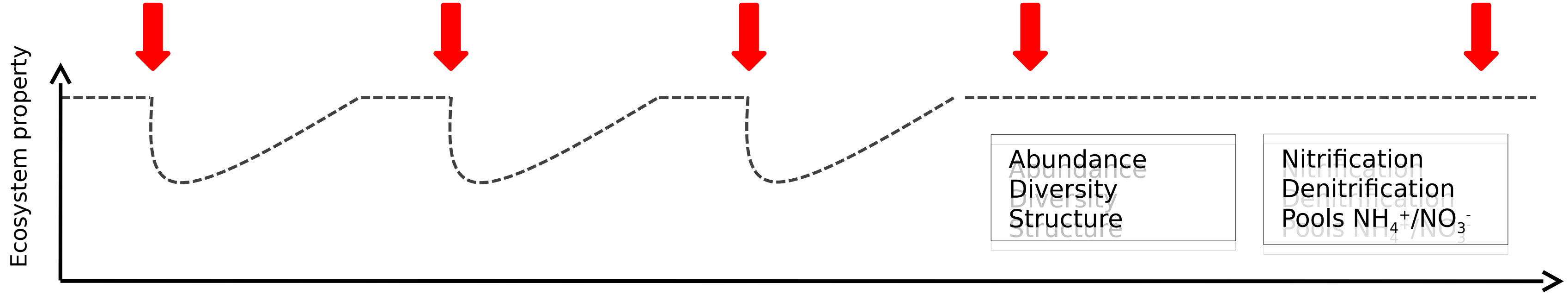

A. List of the 26 studied ecosystem properties and functions.

1. N-related functions

Potential Nitrification Activity (PNA)

Potential Denitrification Activity (PDA)

Potential N_2O emissions

$rN_2O/(rN_2O+rN_2)$ ratio

2. N-pools

NH_4^+

NO_3^-

Ntot

3. Microbial Communities Abundances

q16S

qCrenarcheae

qCrenarcheae/q16S

qAOA and qAOA/qCrenarcheae

qAOB and qAOB/q16S

qnirK and qnirK/q16S

qnirS and qnirS/q16S

qnosZI and qnosZI/q16S

qnosZII and qnosZII/q16S

4. Bacterial Diversity indices

Observed OTUs

Faith’s Phylogenetic Diversity

Simpson’s Reciprocal

Equitability

Supplementary Table 1. Explaining NO_3^- pools changes after repeated disturbances of the same nature.

Repeated disturbance type	**Activities**	**Abundances**	**Diversity indices**
Freeze-Thaw	Potential Denitrification Activity 21.7 %** (-)	q*AOA* 16.2 %** (+)	
Heat	Potential N_2O emissions 41.3 %*** (-)	q*AOB* 12 %*** (-)	Observed Species 4.1 %* (-)
	$rN_2O/r(N_2O+N_2)$ 19.6 %*** (+)	q*nirS* 4.1 %* (-)	
Anoxia	Potential N_2O emissions 32.7 %*** (-)	q*AOB*/q*16S* 16.4 %** (+)	
	Nitrification 8 %* (+)	q*nirS*/q*16S* 9.6 %* (+)	

% of NO_3^- variance explained by each explaining variables is given. The direction of the correlation is given between parentheses.

Significance levels: *** <0.001, ** <0.01, * <0.05

Supplementary Figure 2. Abundance of the 10 most discriminant OTUs between disturbed and control-like microcosms. Panel A represents OTUs whose abundance is greater in control-like microcosms, while panel B represents OTUs that are enriched in disturbed microcosms.

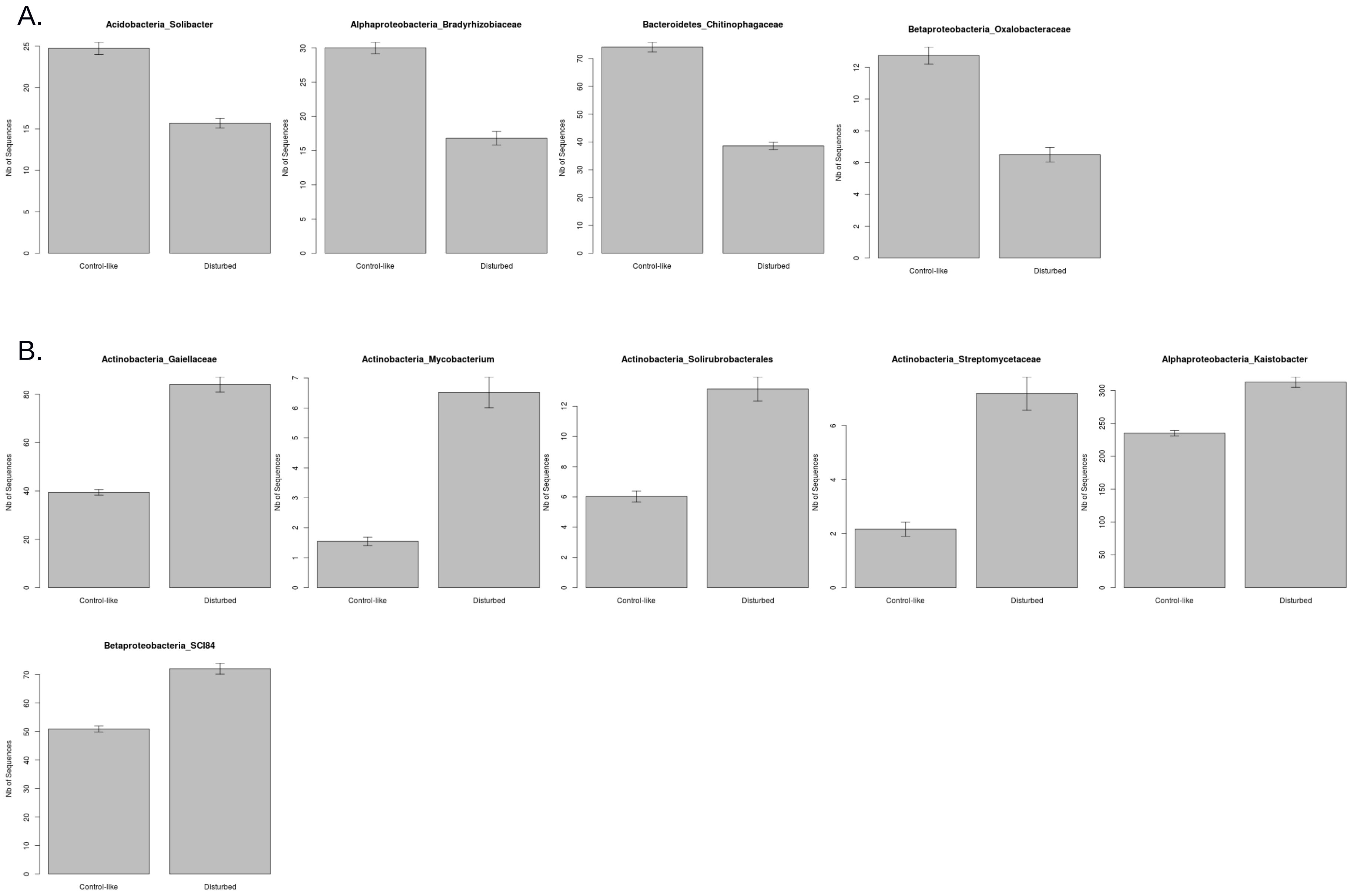

Supplement: Supplementary file 1 [file Data_Sheet_1.PDF]
